# Annotations

**Published:** 1893-01-14

**Authors:** 


### Annotations.

The Drunkards' Deadlock.

The Society for the Study of Inebriety has issued its annual "Proceedings." They well illustrate the wide divergence which exists between knowledge and practice. As a matter of "knowledge," all competent medical men are agreed that the drink habit has its roots in disease; that the disease is usually incurable by the mere efforts of the victim's will; that total abstinence is an effectual cure, and that it ought to be made compulsory upon drunkards under certain reasonable conditions. What now is the state of "practice" in our civilised, religious, and somewhat self-complacent country? The practice is that every drunkard follows his own "sweet will," in the words of the society's annual orator; that he may ruin himself, disgrace and degrade his family, and become an unmitigated nuisance to his neighbours, without let or hindrance from saint or sinner, public functionary or private individual. What is the reason of this ridiculous practical deadlock? It is really a lack of intellectual capacity on the part of the community. We have not intelligence enough to clearly realise the evils of drunkenness, and we have not will enough to apply the obvious and only remedy. But, it may be asked, is it quite safe to convert a drunkard to total abstinence suddenly, and against his will? It is perfectly safe, and, indeed, is the only safety which science knows. The Society for the Study of Inebriety must, we think, hold itself chiefly responsible for the present *impasse*. It has not been successful in framing a practical measure which a reasonable Government can pass into law. It seems to us to be folly, or is it merely want of experience? to expect any Government, overwhelmed as all Governments are with an infinite variety of business, to do the drudgery of framing measures on subjects which only a part of the community consider urgent? If a well-drawn, practical, and generally approved measure were presented to the House of Commons, no doubt it would be passed, both there and in the Lords. It is more than strange that among so many abstaining members of Parliament not one has been found who has succeeded in framing, much less carrying, a reasonable measure through the House. Are we to conclude that total abstainers are, after all, less intelligent and less resolute than those who take a little alcohol?

What Shall we Do with Our Dead?

Something must be done with the dead, whereby they shall be prevented from injuring the living. The Dean of Westminster, Sir Henry Thompson, and other persons who devote their intelligence to this question, were received in deputation by the Home Secretary last week, and, with him, discussed this question of the disposal of the dead. The question is not merely important, it is momentous, and that for three chief reasons: In the first place, England is a small country; in the second, it has an enormous living population; and in the third, it has, consequently, an enormous dead population. To these a fourth may be added—that the population, both living and dead, is concentrated at a strictly limited number of centres. The possibilities of danger under these circumstances are almost infinite. Sir Henry Thompson and his fellow deputationists wish to make one or two sanitary methods of disposal of the dead compulsory throughout the country, and in order to effect this they desire that one central authority shall be created by legislative action which shall have full control and responsibility. The Home Secretary apparently thinks that local authorities have hitherto done fairly well, and that to them we must look in the future. We cannot agree with him in this case. Ordinarily, no doubt, centralisation is to be deprecated, and local self-government is to be developed in every reasonable way. But there are, and must be, exceptions in case of necessity, and this is a case of the sternest possible necessity. We need not rush to cremation, though that would be an useful adjunct, especially in the case of those who die from infectious disease. There is, however, no difficulty in carrying out thoroughly sanitary burial. But in order that this may be done, the choice of cemeteries and other details must be delegated to sanitary experts. The local authorities have neither the knowledge nor the sanitary *esprit* to carry out any such work with uniform instructed intelligence. When we soberly reflect upon such facts as these—that a cemetery of seventeen acres at Tower Hamlets contains 250,000 bodies; that the Brompton Cemetery of twelve acres contains 100,000; and that other indescribable masses of the dead are being huddled together in similar numbers in every large centre of population, we must see that the whole character of the soil cannot but undergo change and poisonous deterioration if burials be not carried out with strict regard to rapid decomposition and sanitary laws. It is a question for the people, not for Home Secretaries and *doctrinaire* politicians. Common sense must be brought to bear upon it; common sense and resolute will: We shall all be poisoned else, and that in our own beautiful England.

The Indispensable "Spade!"

So long as the world endures there will be a never ceasing necessity for "calling a spade a spade." It is not considered polite journalism to dub a professional man "a fool," much less to hint the possibility of his being a "knave." The *Times* has published several letters during the last few days on what its correspondent calls the "New Mesmerism." The letters were admirably written by a skilled exponent of medical science, and were of a nature to carry conviction to minds untrained in the scientific discrimination of facts. Nevertheless, that some hypnotics and hypnotisers are in round terms "fools," and some others "knaves," the letter of Mr. Ernest Hart in the *Times* of January 10th, proves beyond the shadow of doubt. Mr. Hart writes from Paris; he writes as a medical scientist; he gives the results of a personal experience competent in character and duration. Says Mr. Hart: "I was able to carry out at La Charité Hospital itself some very simple test experiments which convinced me that Dr. Luys was the victim, to some extent, of trickery and imposture." But the medical profession and the public are not asked to take the mere word of Mr. Ernest Hart. Among those who were present at one or other of the sittings were Dr. Louis Olivier, directeur de la *Revue Génerale des Sciences*; Dr. Lutaud, editor of the *Journal de Médecin de Paris*; Dr. Sajous, editor of the American *Annual of Medicine*! M. Cremière, of St. Petersburgh, Mr. B. F. C. Costelloe, of London, with others. Such were the witnesses! Here is the briefest possible outline of the results of some of the experiments. Says Mr. Hart: "I need only say here that the whole of the phenomena were reproduced with sham magnets, with substituted figures, with misnamed medicinal substances, with distilled water, and with sham 'suggestion,' opposite suggestion, or none at all. Everyone was able to convince himself that all the results so shown were, without exception, simulated, fictitious, and fraudulent." In other words, the hypnotised persons were impostors, who, however, were not clever enough to discover that they were themselves being imposed upon. Mr. Hart is to publish full details of his experiments But let us appeal to the unsophisticated medical intelligence! Is it not time that all this sensational folly, trickery, and knavery, which has been flaunting itself under the too willing protection of legitimate medicine, should be repudiated, and, when possible, exposed and punished?

